# Herpes simplex virus induces the marked up-regulation of the zinc finger transcriptional factor INSM1, which modulates the expression and localization of the immediate early protein ICP0

**DOI:** 10.1186/1743-422X-8-257

**Published:** 2011-05-25

**Authors:** Maki Kamakura, Fumi Goshima, Chenhong Luo, Hiroshi Kimura, Yukihiro Nishiyama

**Affiliations:** 1 Department of Virology, Nagoya Graduate School of Medicine, 65 Tsurumai-cho, Showa-ku, Nagoya 466-8550, Japan

## Abstract

**Background:**

Herpes simplex viruses (HSVs) rapidly shut off macromolecular synthesis in host cells. In contrast, global microarray analyses have shown that HSV infection markedly up-regulates a number of host cell genes that may play important roles in HSV-host cell interactions. To understand the regulatory mechanisms involved, we initiated studies focusing on the zinc finger transcription factor insulinoma-associated 1 (INSM1), a host cell protein markedly up-regulated by HSV infection.

**Results:**

INSM1 gene expression in HSV-1-infected normal human epidermal keratinocytes increased at least 400-fold 9 h after infection; INSM1 promoter activity was also markedly stimulated. Expression and subcellular localization of the immediate early HSV protein ICP0 was affected by INSM1 expression, and chromatin immunoprecipitation (ChIP) assays revealed binding of INSM1 to the ICP0 promoter. Moreover, the role of INSM1 in HSV-1 infection was further clarified by inhibition of HSV-1 replication by INSM1-specific siRNA.

**Conclusions:**

The results suggest that INSM1 up-regulation plays a positive role in HSV-1 replication, probably by binding to the ICP0 promoter.

## Background

Herpes simplex virus types 1 (HSV-1) and 2 (HSV-2) are large DNA viruses with genomes consisting of at least 74 genes [1], which are classified into four groups with respect to their order of expression on the entry of HSV into the host cell. Immediate early (IE) genes are transcribed without prior viral protein synthesis. Early genes are expressed before the onset of viral DNA synthesis and require IE gene expression. Expression of delayed early genes is partially dependent on viral DNA synthesis, but that of late or true late genes is completely dependent on viral DNA synthesis. The cascade of HSV gene expression is tightly regulated by viral and cellular factors [2-6].

HSV infection markedly affects expression of host cell genes. The HSV genome encodes a virion-associated endonuclease UL41 that degrades viral and cellular mRNA early in infection. The IE protein ICP27 also inhibits cellular gene expression by blocking mRNA splicing [7]. Although the high level of viral transcription appears to overcome the effect of these proteins, host cell protein synthesis is strongly suppressed early in HSV infection. However, microarray analysis has shown that HSV-infected cells express high levels of a significant number of cellular genes [8]. We have shown that transcript levels of the cellular genes ZSCAN4, ZNF342, and HBA2 increased by more than 100-fold in both HSV-1- and HSV-2-infected HEp-2 cells [8]. Although whether enhanced expression of these three genes at the transcriptional level corresponds to increased expression of their gene products is unclear, such marked host cell responses may reveal novel regulatory mechanisms involved in HSV replication. 

Cells of the developing central and peripheral nervous system as well as endocrine cells of the developing pancreas and intestine express insulinoma-associated 1 (INSM1), a zinc finger transcription factor [9]. More specifically, INSM1 gene expression is highly restricted to fetal pancreatic and brain tissues [10-14]. Since INSM1 is also highly expressed in tumors of neuroendocrine origin, its promoter could serve as a tumor-specific target for gene therapy for neuroendocrine tumors [15-17]. Recent studies have shown that INSM1 is a crucial component of the transcriptional network that controls differentiation of the sympatho-adrenal lineage [18], and that INSM1 is involved in the generation and expansion of basal progenitors in the developing neocortex [19].

In the present study, we found that INSM1 gene expression was markedly stimulated by HSV-1 and HSV-2 infections of normal human epidermal keratinocytes (NHEK) and HaCaT cells. We also report the effects of INSM1 on expression and distribution of the IE protein ICP0 and a possible role of INSM1 in HSV-1 replication.

## Results

### Microarray analysis of cellular transcriptional responses to HSV-1 and HSV-2 infections

**Figure 1:**
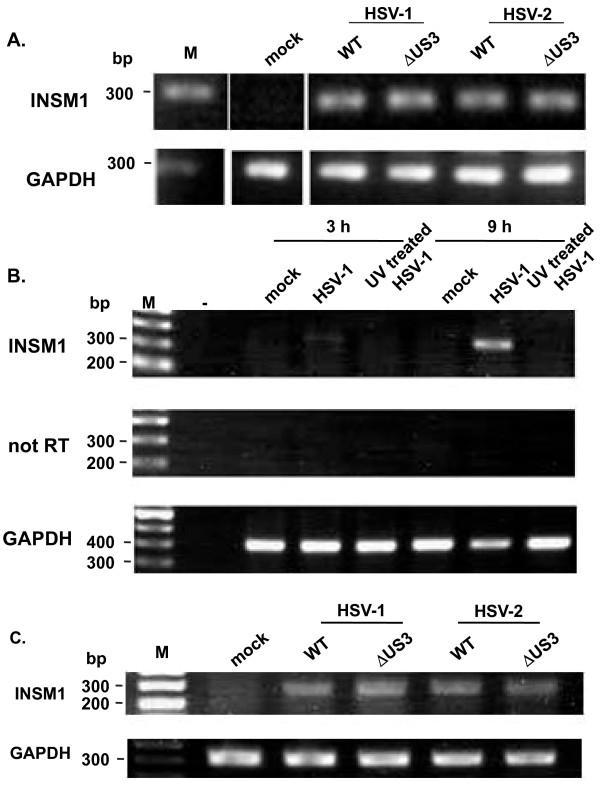
**INSM1 expression in HSV-infected cells**. **(A) **NHEK cells were infected with wild-type (WT) HSV-1, WT HSV-2, and their US3 deletion mutants (multiplicity of infection, MOI = 3), and total RNA was extracted 9 h after infection. INSM1 mRNA was amplified by RT-PCR as described in Methods. GAPDH was used as a control. Data represent two independent experiments. **(B) **HaCaT cells were infected with HSV-1 or UV-inactivated HSV-1 (MOI = 3), and total RNA was extracted for RT-PCR at 3 and 9 h after infection. **(C) **HEp-2 cells were infected with WT HSV-1, WT HSV-2, and their US3 deletion mutants (MOI = 3), and total RNA was extracted for RT-PCR at 9 h after infection.

We previously reported that HSV-1 and HSV-2 infections markedly increased mRNA levels of specific cellular genes in HEp-2 cells [8]. Since the HEp-2 cell line is derived from a tumor, responses of HEp-2 cells to HSV infections may differ from those of non-transformed cells. Therefore, we performed global microarray analysis of NHEK cells that were mock infected or infected with wild-type (WT) HSV-1, WT HSV-2, and their US3 mutants. While US3 is not essential for viral replication in vitro, the protein kinases encoded by the US3 genes of HSV-1 and HSV-2 have been shown to play important roles in various aspects of viral replication and pathogenicity, including regulation of apoptosis and signal transduction and virion maturation [20-24]. We thus examined the transcriptional responses of cells infected with the US3 mutants. Table 1 shows cellular genes whose mRNA levels increased by at least 4-fold 9 h after infection. Among the 54,765 probe sets examined, levels of 189 transcripts increased by at least 4-fold in infected NHEK cells and those of 108 transcripts increased in common in both NHEK and HEp-2 cells. In NHEK cells, INSM1 expression was always highly up-regulated in all cases. Our microarray analysis showed that the level of INSM1 mRNAs increased by at least 400-fold 9 h after infection in HSV-infected cells compared with mock-infected cells. Although the extent of increase was higher in US3 mutant-infected cells than in WT-infected cells, the mechanism remains unclear. The marked up-regulation of INSM1 by HSV infections was confirmed by reverse transcription (RT)-PCR analysis of NHEK, HaCaT, and HEp-2 cells (Figure 1). Moreover, we found that such up-regulation was not induced by UV-inactivated HSV-1 (Figure 1B). The INSM1 gene, similar to most HSV genes, lacks introns [25]. INSM1 has recently been shown to bind cyclin D1 and play an important role in switching cells between cellular proliferation and differentiation pathways [26]. Therefore, we focused here on HSV-induced INSM1 up-regulation.

**Table 1:** Human genes induced by HSV infection of NHEK cells at 9h after infection

Representative Public ID ^a^	Gene Symbol	Gene Title	Fold change ^b^				Gene Ontology Biological Process
			HSV-1		HSV-2		
			WT	ΔUS3	WT	ΔUS3	
**NM_005083**	***U2AF1L1***	**U2 small nuclear RNA auxillary factor 1-like 1**	**4.0**	**13.0**	**5.3**	**7.5**	**---**
**AV712346**	***REEP5***	**Receptor accessory protein 5**	**9.8**	**11.3**	**11.3**	**9.8**	**---**
**AK091308**	***NARG1***	**NMDA receptor regulated 1**	**26.0**	**59.7**	**36.8**	**19.7**	**---**
**AW613101**	***KCNQ1OT1***	**KCNQ1 overlapping transcript 1**	**32.0**	**45.3**	**42.2**	**78.8**	**---**
**AW014728**	***FLJ39575***	**hypothetical protein FLJ39575**	**90.5**	**111.4**	**78.8**	**207.9**	**---**
**AL359567**	***IGSF4***	**Immunoglobulin superfamily, member 4**	**8.0**	**17.1**	**10.6**	**7.5**	**immune response**
**NM_000594**	***TNF***	**tumor necrosis factor (TNF superfamily, member 2)**	**21.1**	**34.3**	**8.6**	**11.3**	**immune response**
**AW135003**	***API5***	**Apoptosis inhibitor 5**	**4.3**	**6.1**	**4.6**	**5.7**	**apoptosis**
**AW511239**	***RHOT1***	**Ras homolog gene family, member T1**	**4.9**	**6.1**	**6.5**	**8.6**	**apoptosis**
**AA005430**	***CROP***	**Cisplatin resistance-associated overexpressed protein**	**7.0**	**13.0**	**13.0**	**9.8**	**apoptosis**
**NM_006538**	***BCL2L11***	**BCL2-like 11 (apoptosis facilitator)**	**39.4**	**29.9**	**19.7**	**39.4**	**apoptosis**
**NM_006910**	***RBBP6***	**retinoblastoma binding protein 6**	**4.0**	**6.5**	**6.1**	**6.1**	**cell cycle**
**NM_000076**	***CDKN1C***	**cyclin-dependent kinase inhibitor 1C (p57, Kip2)**	**4.9**	**5.7**	**8.0**	**7.0**	**cell cycle**
**X07868**	***IGF2***	**insulin-like growth factor 2 (somatomedin A)**	**6.1**	**7.0**	**5.7**	**7.5**	**cell cycle**
**NM_015675**	***GADD45B***	**growth arrest and DNA-damage-inducible, beta**	**12.1**	**14.9**	**14.9**	**34.3**	**cell cycle**
**NM_003914**	***CCNA1***	**cyclin A1**	**13.0**	**21.1**	**13.0**	**14.9**	**cell cycle**
**BE348555**	***PDZK1***	**PDZ domain containing 1**	**4.0**	**9.2**	**4.6**	**6.5**	**signal transduction**
**NM_005204**	***MAP3K8***	**mitogen-activated protein kinase kinase kinase 8**	**4.0**	**12.1**	**4.6**	**6.5**	**signal transduction**
**NM_003328**	***TXK***	**TXK tyrosine kinase**	**5.7**	**5.3**	**9.8**	**10.6**	**signal transduction**
**NM_014823**	***WNK1***	**WNK lysine deficient protein kinase 1**	**6.1**	**5.3**	**9.8**	**7.0**	**signal transduction**
**AI684439**	***GRAP***	**GRB2-related adaptor protein**	**13.9**	**8.6**	**6.1**	**16.0**	**signal transduction**
**NM_018390**	***PLCXD1***	**phosphatidylinositol-specific phospholipase C, X domain containing 1**	**11.3**	**34.3**	**17.1**	**19.7**	**signal transduction**
**AF069506**	***RASD1***	**RAS, dexamethasone-induced 1**	**111.4**	**104.0**	**111.4**	**207.9**	**signal transduction**
**NM_004692**	***INA***	**internexin neuronal intermediate filament protein, alpha**	**6.5**	**9.8**	**4.6**	**4.6**	**cell adhesion and cytoskeleton**
**AI668588**	***SPTAN1***	**Spectrin, alpha, non-erythrocytic 1 (alpha-fodrin)**	**16.0**	**27.9**	**26.0**	**26.0**	**cell adhesion and cyctoskeleton**
**NM_005382**	***NEFM***	**neurofilament, medium polypeptide 150kDa**	**29.9**	**36.8**	**26.0**	**55.7**	**cell adhesion and cytoskeleton**
**U26662**	***NPTX2***	**neuronal pentraxin II**	**45.3**	**52.0**	**32.0**	**34.3**	**cell communication**
**NM_002105**	***H2AFX***	**H2A histone family, member X**	**10.6**	**13.9**	**7.5**	**13.0**	**DNA / chromosome**
**NM_003655**	***CBX4***	**chromobox homolog 4 (Pc class homolog, Drosophila)**	**24.3**	**29.9**	**14.9**	**36.8**	**DNA/chromosome**
**AL832081**	***ZNF131***	**Zinc finger protein 131**	**4.3**	**7.0**	**6.1**	**9.8**	**transcription**
**T79183**	***JAZF1***	**JAZF zinc finger 1**	**5.7**	**4.9**	**7.5**	**8.6**	**transcription**
**D42040**	***BRD2***	**bromodomain containing 2**	**6.1**	**9.2**	**6.1**	**8.6**	**transcription**
**NM_001452**	***FOXF2***	**forkhead box F2**	**6.5**	**14.9**	**11.3**	**16.0**	**transcription**
**NM_005904**	***SMAD7***	**SMAD family member 7**	**7.0**	**9.8**	**7.0**	**12.1**	**transcription**
**AF193855**	***ZIC2***	**Zic family member 2 (odd-paired homolog, Drosophila)**	**13.0**	**55.7**	**34.3**	**68.6**	**transcription**
**NM_001674**	***ATF3***	**activating transcription factor 3**	**13.9**	**16.0**	**36.8**	**26.0**	**transcription**
**AI459175**	***KLF3***	**Kruppel-like factor 3 (basic)**	**14.9**	**18.4**	**16.0**	**14.9**	**transcription**
**NM_004405**	***DLX2***	**distal-less homeobox 2**	**17.1**	**24.3**	**13.9**	**42.2**	**transcription**
**AW274658**	***ING1***	**Inhibitor of growth family, member 1**	**19.7**	**12.1**	**10.6**	**24.3**	**transcription**
**NM_002166**	***ID2***	**inhibitor of DNA binding 2, dominant negative helix-loop-helix protein**	**24.3**	**22.6**	**19.7**	**42.2**	**transcription**
**NM_002448**	***MSX1***	**msh homeobox 1**	**59.7**	**84.4**	**48.5**	**128.0**	**transcription**
**NM_152677**	***ZSCAN4***	**zinc finger and SCAN domain containing 4**	**97.0**	**362.0**	**55.7**	**147.0**	**transcription**
**AA761573**	***ZNF342***	**zinc finger protein 342**	**128.0**	**256.0**	**111.4**	**294.1**	**transcription**
**NM_002196**	***INSM1***	**insulinoma-associated 1**	**445.7**	**776.0**	**955.4**	**1782.9**	**transcription**
**AI906424**	***HNRPM***	**Heterogeneous nuclear ribonucleoprotein M**	**4.3**	**5.7**	**6.5**	**11.3**	**RNA / translation**
**AI445255**	***SFRS3***	**Splicing factor, arginine/serine-rich 3**	**13.0**	**14.9**	**14.9**	**21.1**	**RNA / translation**
**R17062**	***PABPC1***	**Poly(A) binding protein, cytoplasmic 1**	**32.0**	**39.4**	**48.5**	**90.5**	**RNA / translation**
**BF195994**	***PIAS2***	**Protein inhibitor of activated STAT, 2**	**4.3**	**5.7**	**4.6**	**9.2**	**protein folding and modification**
**U56725**	***HSPA2***	**heat shock 70kDa protein 2**	**5.7**	**8.6**	**4.0**	**5.7**	**protein folding and modification**
**AU158573**	***ABHD5***	**Abhydrolase domain containing 5**	**13.0**	**18.4**	**11.3**	**19.7**	**protein folding and modification**
**NM_000104**	***CYP1B1***	**cytochrome P450, family 1, subfamily B, polypeptide 1**	**6.1**	**6.5**	**13.0**	**18.4**	**development**
**NM_001529**	***HHEX***	**homeobox, hematopoietically expressed**	**17.1**	**48.5**	**97.0**	**68.6**	**development**
**NM_002405**	***MFNG***	**MFNG O-fucosylpeptide 3-beta-N-acetylglucosaminyltransferase**	**4.0**	**8.0**	**4.6**	**8.6**	**developmental process**
**NM_000361**	***THBD***	**thrombomodulin**	**9.8**	**11.3**	**5.7**	**8.6**	**biological regulation**
**NM_000499**	***CYP1A1***	**cytochrome P450, family 1, subfamily A, polypeptide 1**	**4.3**	**4.9**	**5.3**	**7.5**	**cellular metabolism**
**AI625747**	***ADRB1***	**adrenergic, beta-1-, receptor**	**6.1**	**18.4**	**7.5**	**10.6**	**cellular metabolism**
**NM_003895**	***SYNJ1***	**synaptojanin 1**	**6.5**	**13.0**	**7.5**	**11.3**	**cellular metabolism**
**NM_000558**	***HBA1***	**hemoglobin, alpha 1**	**55.7**	**84.4**	**34.3**	**137.2**	**transport**

^a ^GeneBank accession No. corresponds to sequence to which the Affymetrix U133 plus 2 probe set was designed.

^b ^Fold change was calculated by comparison to values obtained from the mock-infected cells.

### HSV infection stimulates human INSM1 promoter activity

**Figure 2:**
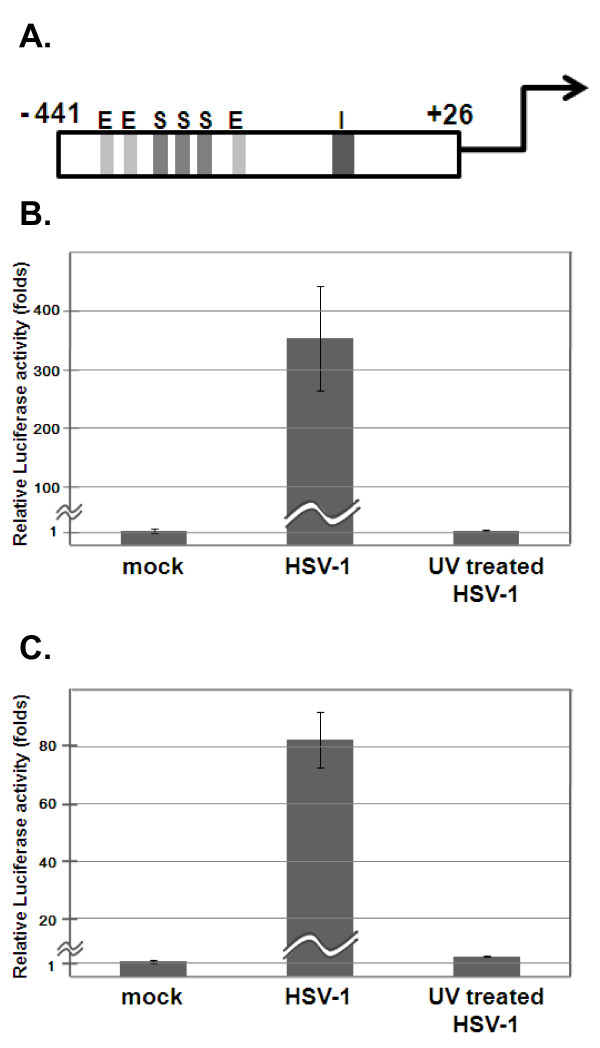
**Effect of HSV infection on INSM1 promoter-directed luciferase activities**. **(A) **Schematic representation of the INSM1 promoter region from position -441 to +26. Predicted transcription factor binding sites are as follows: E (E box, 5′-CATTTG-3′, 5′-CACGTG-3′, and 5′-CATCTG-3′), S (SP1-binding site, 5′-GGGGCCGGGC-3′ and 5′-CGGGCGGGC-3′), and I (INSM1-binding site, 5′-CTCCAGGGGAAGC-3′). **(B **and **C) **Luciferase reporter assays. HaCaT **(B) **and HEp-2 **(C) **cells were infected with WT HSV-1 and UV-inactivated HSV-1 (MOI = 3) and harvested 9 h after infection. Luciferase activity was measured as described in Methods. The results are shown as means ± standard deviation (SD).

To investigate the effect of HSV infection on INSM1 promoter-dependent expression, reporter gene assays were performed. Figure 2A shows a schematic representation of human INSM1 promoter region (-441 to +26 bp), which was amplified by PCR using DNA extracted from HEp-2 cells, confirmed by sequence analysis, and cloned into the pGL3 luciferase reporter vector. HaCaT and HEp-2 cells were transfected with this construct or empty vector DNA and then infected with WT HSV-1 at a multiplicity of infection (MOI) of 3 plaque-forming units (PFU)/cell 24 h after transfection. The cells were collected 9 h after infection. INSM1 promoter activity increased approximately 350-fold in infected HaCaT cells (Figure 2B) and 80-fold in infected HEp-2 cells (Figure 2C). In infected HEp-2 and HaCaT cells, luciferase activity rapidly increased until 12 h after infection (data not shown). Extracts prepared from cells infected with UV-inactivated HSV-1 failed to support INSM1 promoter activity. These observations indicate that HSV infection activates the INSM1 promoter.

### Subcellular localization of human full-length INSM1 (INSM1-FL) and mutant proteins

**Figure 3:**
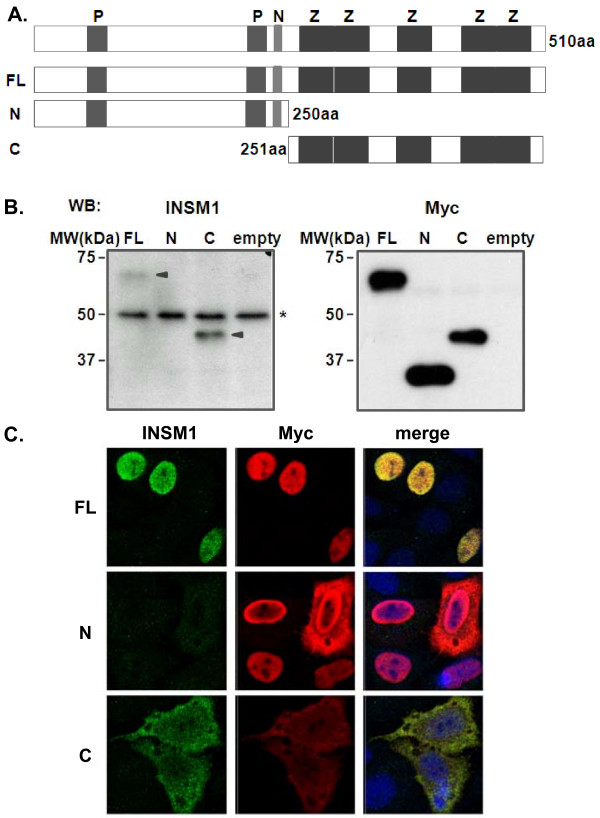
**Expression of INSM1 and INSM1 deletion mutants**. **(A) **Schematic diagram of INSM1 domain structure. INSM1 has two proline-rich domains (P, amino acid residues 43-58 and 75-84), one NLS (N, amino acids residues 221-246) and five C2H2 zinc finger motifs (Z). HEp-2 cells were transfected with FL or deletion mutant INSM1 expression vectors and incubated for 24-48 h prior to analysis by Western blotting **(B) **and confocal microscopy **(C)**. The positions of mutant proteins detected by polyclonal antibodies are shown. Anti-Myc rabbit polyclonal antibody **(B, *right*) **and anti-INSM1 rabbit polyclonal antibody **(B, *left*) **were used to detect full-length and mutant proteins, respectively. The asterisk indicates a non-specific band.

We next constructed human INSM1 expression vectors to investigate the effects of INSM1 on HSV replication. Human INSM1 is a 58-kDa protein that contains five zinc finger motifs in its C-terminal domain (Figure 3A). The N-terminal domain contains a putative nuclear localization signal (NLS) and proline-rich domains that interact with cyclin D1 [26]. Thus, the expression vectors of INSM1 N- and C-terminal domains were constructed and used along with a INSM1-FL construct to transfect HEp-2 cells. The cells were subjected to Western blot analysis 24 h after transfection. As determined by Western blot analysis, the anti-INSM1 antibody raised against the human INSM1 C-terminal peptides (NP_002187, amino acid residues 393-442) reacted weakly with a 60-kDa band in INSM1-FL-transfected cells and a 40-kDa band in INSM1-C-transfected cells (Figure 3B). Both bands were easily detected by the anti-Myc antibody because of the Myc-tag epitope expressed by the constructs. Although a 50-kDa band was detected in all cases, it appeared to be non-specific because the apparent molecular size was smaller than the expected size of human INSM1-FL and the band intensity did not change after HSV infections.

Transfected cells were fixed, reacted with anti-human INSM1 and anti-Myc antibodies, and examined by confocal laser microscopy (Figure 3C). In INSM1-FL-transfected cells, specific INSM1 fluorescence was detected exclusively in the nucleus. In INSM1-N-transfected cells, no specific fluorescence was detected when examined with the anti-INSM1 antibody. However, immunofluorescence staining using the anti-Myc antibody revealed that INSM1-N was localized in the nucleus in a majority of cells and in the cytoplasm of some cells. In contrast, INSM1-C that lacked a putative NLS predominantly localized in the cytoplasm, as expected.

### Effect of INSM1 and its mutant proteins on expression and subcellular localization of ICP0

**Figure 4:**
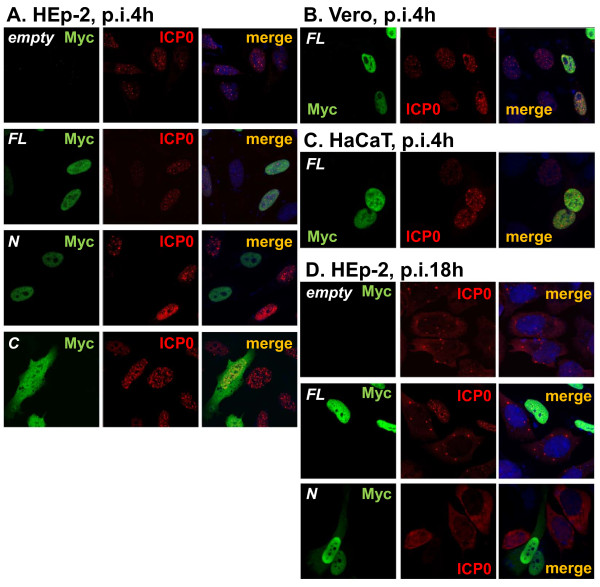
**Expression and localization of ICP0 in INSM1-expressing cells**. HEp-2 **(A **and **D)**, Vero **(B)**, and HaCaT **(C) **cells were transfected with plasmids encoding Myc-tagged full-length INSM1 (INSM1-FL) and deletion mutants (N and C). At 20-24 h after transfection, cells were infected with HSV-1(F) (MOI = 3), fixed at 4 h **(A, B **and **C) **or 18 h **(D) **after infection, and reacted with anti-Myc and anti-ICP0 antibodies as described in Methods.

INSM1-binding sequences have been identified as T^G^/T^C^/T^C^/T^T^/AGGGG^G^/TC^G^/A [27]. To identify consensus sequences for INSM1 binding in HSV-1 (17^+^, X14112) and HSV-2 (HG52, Z86099) genomes, their complete genomic sequences were analyzed using GENETYX version 8 (Software Development Co., Tokyo, Japan). The results showed that both HSV-1 and HSV-2 genomes contain several INSM1-binding sequences, but only one sequence was present in the upstream promoter region of the specific gene ICP0, suggesting that ICP0 could be an INSM1 target. Therefore, we examined the effect of INSM1 on expression and localization of ICP0 in HSV-1-infected cells. The cells were transfected with each INSM1 expression vector and infected with HSV-1 at MOI of 3 PFU/cell 24 h after transfection. The cells were then fixed at appropriate times after infection and processed for visualization by immunofluorescent confocal laser microscopy (Figure 4). ICP0 signals were detected as dots in the nucleus 4 h after infection in empty vector-transfected cells, as expected (Figure 4A). The distribution pattern of ICP0 in INSM1-FL-expressing cells was similar to that in mock-transfected cells, but the intensity of ICP0-specific fluorescence appeared to be higher in INSM1-FL-expressing cells than in non-expressing cells. Such augmented expression of ICP0 in INSM1-FL-expressing cells was more clearly observed in Vero and HaCaT cells (Figure 4B and 4C). Interestingly, ICP0 expression was strongly suppressed in most INSM1-N-expressing cells in contrast to INSM1-C-expressing cells (Figure 4A). Nuclear ICP0 is known to translocate to the cytoplasm late in HSV infection [28]. In fact, ICP0 was predominantly localizaed in the cytoplasm of mock-transfected cells 18 h after infection (Figure 4D). In INSM1-FL-expressing cells, however, ICP0 remained in the nucleus, indicating that INSM1 can inhibit the translocation of ICP0 to the cytoplasm late in infection.

**Figure 5:**
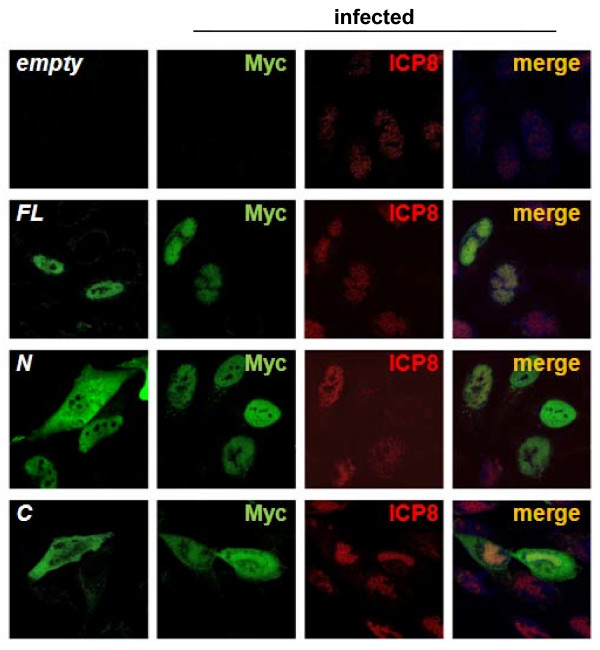
**Subcellular localization of ICP8 in INSM1-expressing cells**. HEp-2 cells were transfected with plasmids encoding Myc-tagged INSM1-FL and deletion mutants (N and C). At 20-24 h after transfection, cells were infected with HSV-1(F) (MOI = 3), fixed 18 h after infection, and reacted with anti-Myc and anti-ICP8 antibodies as described in Methods.

We also examined the effect of INSM1 on expression and subcellular localization of the single-strand DNA-binding protein ICP8 (Figure 5). No evidence showed that INSM1-FL or INSM1-C expression affected ICP8 expression, but ICP8 expression appeared to be suppressed in INSM1-N-expressing cells. In HSV-1-infected cells, INSM1-FL localized to the replication compartment late in infection. Furthermore, subcellular localization of INSM1-N and INSM1-C was found to be affected by HSV-1 infection, with both mutant proteins co-localizing with ICP8 in the nucleus.

These observations suggest that INSM1 expression enhanced ICP0 expression early in infection but inhibited the translocation of ICP0 to the cytoplasm late in infection, and that ICP0 expression was suppressed by INSM1-N even during the late phase. However, we have so far failed to show a significant difference in ICP0 expression between INSM1-transfected or mock-transfected cells by Western blot analysis, probably because of low transfection efficiency.

### Effect of INSM1 on ICP0 promoter activity

**Figure 6:**
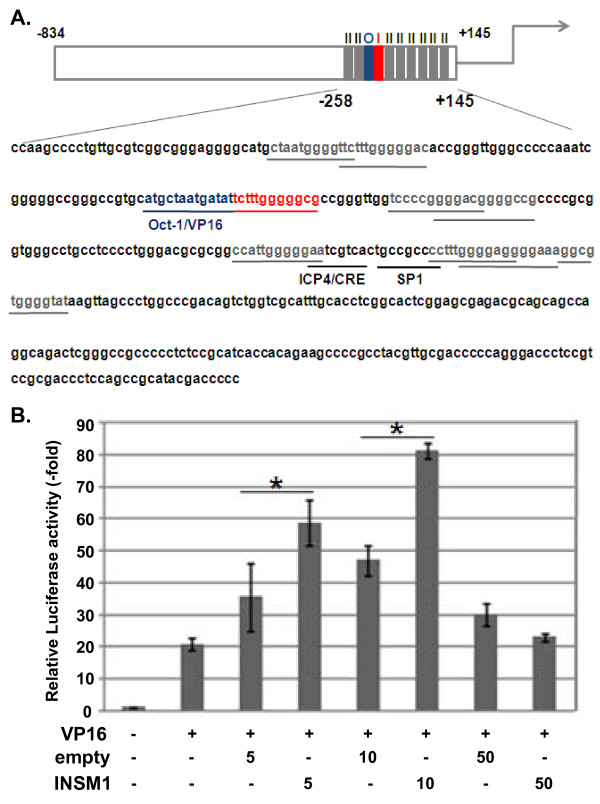
**Effect of HSV infection on ICP0 promoter-derived luciferase activities**. **(A) **Graphical representation of the nucleotide sequences of the ICP0 promoter region from position -834 to +145. The HSV-1 ICP0 promoter has several potential INSM1-binding sites (red, INSM1-binding consensus site, 5′-TCCCCGGGGACG-3′; gray, INSM1-like binding site, 5′-TNNNNGGGGNCN-3′). The binding sites of well-known transcription factors are as follows: blue, Oct-1/VP16 binding site, 5′-ATGCTAATGATAT-3′. **(B) **HEp-2 cells were transfected with plasmid DNAs containing reporter and effector genes. Luciferase activity was measured as described in Methods. The results are shown as means ± SD, *P < 0.05 (Student's *t *test) relative to the empty vector (EF).

As described above, the ICP0 promoter contains the INSM1-binding consensus sequence T^G^/T^C^/T^C^/T^T^/AGGGG^G^/TC^G^/A [27] and several potential INSM1-binding sequences, TNNNNGGGGNCN (Figure 6A). In addition, our observations showed that INSM1 expression significantly modulated ICP0 expression in HSV-1-infected cells. Therefore, we wanted to determine the effect of INSM1 on the ICP0 promoter by luciferase reporter gene assays. Because the ICP0 promoter contains binding sites for the Oct-1 protein complex containing host cell factor (HCF), and the HSV virion-associated transactivator VP16 [29,30] and the transcription of the ICP0 gene is highly activated by VP16 [31], we examined the effect of INSM1 on ICP0 promoter activity in the presence of VP16. In fact, the ICP0 promoter exhibited extremely weak activity in the absence of VP16 when examined by reporter assays in HEp-2 cells (data not shown); therefore, the effect of INSM1 was measured under conditions in which cells were co-transfected with a VP16 expression vector (Figure 6B). Luciferase activity was enhanced approximately 20-fold by the VP16 expression vector and was further augmented by INSM1 expression although the addition of an empty vector also increased the luciferase activity to some extent. The difference between the INSM1 expression vector and empty vector was statistically significant and repeatedly observed in three independent experiments. However, the addition of increased amounts of the INSM1 expression vector suppressed luciferase expression. A similar trend was observed in HEp-2 and HaCaT cells. These results indicate that the ICP0 promoter can be activated by INSM1 under specific conditions.

### INSM1 binds the ICP0 promoter

**Figure 7:**
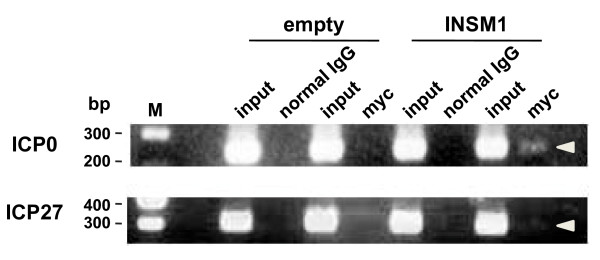
**Binding of INSM1 to the ICP0 promoter**. ChIP assays were performed as described in Methods. INSM1-DNA complexes were incubated with the anti-Myc antibody or normal rabbit IgG. The precipitated and genomic DNAs (input) were subjected to PCR amplification using primers specific for both ICP0 and ICP27 promoters. Data represent two independent experiments.

We performed ChIP assays to determine whether INSM1 binds the ICP0 promoter. Thus, HEp-2 cells were transfected with empty and INSM1-FL vectors, infected with HSV-1(F) 48 h after transfection, fixed with formaldehyde 7 h after infection, and processed for ChIP assays (see Methods). Chromatin solution was incubated with the anti-Myc antibody or control IgG, and the immunoprecipitated complexes were analyzed by PCR using ICP0 promoter-specific primers and ICP27 promoter-specific primers as controls. Figure 7 shows that ICP0 promoter sequences were amplified by chromatin complexes precipitated by anti-Myc antibodies, but not by normal IgG. The ICP27 promoter sequence was not detectably amplified in either case. Therefore, these results indicate that INSM1 selectively binds the ICP0 promoter.

### INSM1-specific siRNA inhibits HSV replication

**Figure 8:**
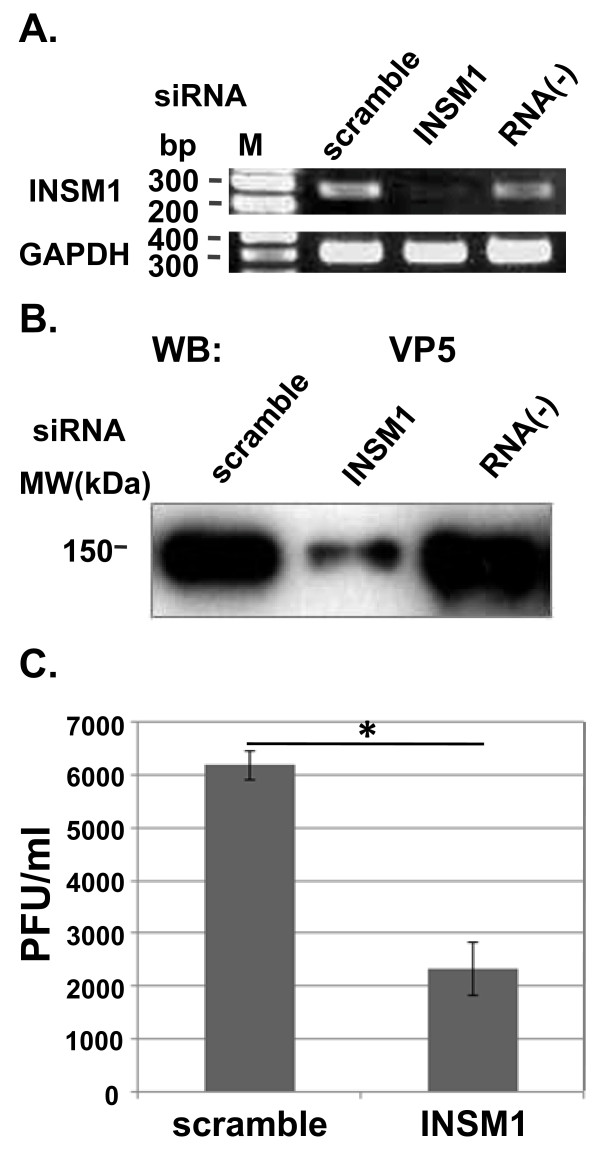
**Effect of INSM1-specific siRNA on HSV-1 replication**. HEp-2 cells were transfected with siRNA specific for human INSM1 and a scrambled oligomer. At 24 h after transfection, HEp-2 cells were infected with HSV-1(F) (MOI = 1), and total RNA was extracted 6 h after infection. INSM1 mRNA was amplified by RT-PCR as described in Methods. GAPDH was used as a control **(A)**. At 48 h after transfection, cells were infected with HSV-1(F) (MOI = 0.01), and the amount of the major capsid protein VP5 **(B) **and infectivity **(C) **were measured at 48 h after infection by Western blotting and plaque assays, respectively. Results of viral titers are shown as mean ± SD. *P < 0.05 (Student's *t *test) relative to the scrambled control oligomer.

The above observations lead us to conclude that INSM1 expression enhances HSV-1 replication. To better support this conclusion, the effect of INSM1 knockdown on the efficiency of viral replication was examined using siRNA technology. INSM1-specific siRNA markedly reduced the level of INSM1 mRNA, but scramble siRNA did not (Figure 8A). Cells were mock-transfected or transfected with either scrambled siRNA or siRNA specific for INSM1, and then infected with HSV-1 at MOI of 0.01 PFU/cell 48 h after transfection. At 48 h after infection, culture medium was harvested and assayed for viral infectivity and subjected to Western blot analysis. As shown in Figure 8B and 8C, pretreatment of cells with INSM1-specific siRNA suppressed HSV-1 replication as well as the production of the major capsid protein VP5. These results indicate that INSM1 supports HSV-1 replication in HEp-2 cells.

## Discussion

The transcription factor INSM1 plays an important role in developing neuroendocrine tissues and the nervous system in mammals [9]. The expression pattern of INSM1 is restricted to the fetal tissues and silenced in adult tissues [9]. However, the INSM1 gene is known to be reactivated in neuroendocrine tumors [15,32]. Our present study shows that transcriptional expression of the INSM1 gene was markedly up-regulated by HSV-1 or HSV-2 infection. This marked increase was observed in normal and human tumor cell lines. In addition, we employed an INSM1 promoter-driven luciferase expression vector to confirm that INSM1 promoter activity was markedly elevated by infection with HSV and not with UV-inactivated HSV, strongly suggesting that HSV gene expression is necessary for activating the INSM1 promoter. Since an immediate early gene product, ICP4 is well known to activate the expression of cellular genes, it is a possible candidate for a transactivator of the INSM1 gene. Previous studies have shown that ICP4 forms a tripartite complex with TFIIB and TATA binding protein (TBP) on DNA and interacts with TBP associated factor 250 (TAF250) [33,34]. ICP4 promotes the formation of transcription preinitiation complex by enhancing TFIID binding to the promoter [35]. Therefore, it is possible that ICP4 could transactivate INSM1. VP16 could also be involved in the activation of the ISNM1 promoter. However, the exact mechanism by which HSV infection activates the INSM1 promoter remains to be determined.

The INSM1 gene, similar to most HSV genes, lacks introns [25]. The IE protein ICP27 inhibits host cell mRNA splicing, resulting in the accumulation of unspliced transcripts in the nucleus and mediates RNA export of intronless mRNA. Therefore, we expected that the INSM1 gene product could be detected in HSV-infected cells but failed to detect it, probably because of the low specificity and reactivity of the anti-INSM1 antibody used. Currently, whether up-regulation of INSM1 mRNA in HSV-infected cells result in the production of functional INSM1 is unclear.

We constructed the expression vectors of INSM1-FL and its N- and C-terminal domains fused in the frame with the Myc-tag epitope. Using these expression vectors, we observed that INSM1 expression enhanced ICP0 expression early in infection. Nucleotide sequence analysis showed that the upstream promoter region of ICP0 contained an INSM1-binding consensus sequence and several potential INSM1-binding sequences, suggesting that INSM1 directly exerted its effect on ICP0 expression by binding its promoter region. In fact, our reporter gene assays showed that INSM1 significantly stimulated the ICP0 promoter in the presence of the virion-associated transactivator VP16. However, the addition of increased amounts of the INSM1 expression vector suppressed luciferase expression. When added at high concentrations, VP16 might attract Oct-1 and HCF to vector sites instead of ICP0 promoter. It seems likely that some kind of swamping would be induced in the expression vectors. In this study, we also showed that INSM1 binds the ICP0 promoter but not the ICP27 promoter. Taken together, our results strongly suggest that INSM1 regulates ICP0 expression by binding the ICP0 promoter. However, INSM1 could also indirectly modulate ICP0 expression by inducing expression of cellular factors involved in ICP0 expression.

Based on the amino acid sequences, INSM1 can be functionally divided into N- and C-terminal regions [9]. The C-terminal region of human INSM1 contains five zinc finger domains that mediate DNA binding activity. The N-terminal region contains a putative NLS and two proline-rich domains that can serve as protein-protein interacting domains. In fact, the proline-rich N-terminal portion of INSM1 has recently been shown to specifically bind cyclin D1, a key cell cycle regulator, and that through this interaction, INSM1 interrupts the interaction between cyclin D1 and cyclin-dependent kinase 4 (CDK4), which subsequently inhibits Rb phosphorylation [26]. HSV induces Rb hypophosphorylation in infected cells, and it has been suggested that the inhibition of although the precise mechanism remains unclear [36]. We therefore postulate that INSM1 can directly and indirectly modulate transcriptional expression of ICP0.

Subcellular localization of ICP0 was markedly affected by INSM1 overexpression late in infection. During the IE phase of infection, ICP0, a RING finger E3 ubiquitin ligase, is exclusively nuclear and acts as a nuclear regulator of HSV mRNA synthesis [37]. However, ICP0 translocates to the cytoplasm during the early phase [28], and a recent study showed that cytoplasmic ICP0 plays a role in virion maturation and/or egress by dismantling the host cell's microtubules networks [38]. Our observations showed that INSM1 inhibits the translocation of ICP0 to the cytoplasm. Recently, Kalamavoki and Riozman showed that inhibition of CDK4 kinase activity inhibits the translocation of ICP0 from the nucleus to the cytoplasm in infected cells [39]. INSM1 can indirectly inhibit CDK4 function through its interaction with cyclin D1, and therefore, the N-terminal proline-rich domains of INSM1 may be involved in this phenomenon. In the late stage of the replication cycle, INSM1 may inhibit virus replication. However, this effect may be an artifact caused by INSM1 overexpression in transfected cells.

## Conclusions

In summary, our study clearly demonstrates that HSV infection up-regulates the expression of INSM1 which can regulate expression and localization of the IE protein ICP0. Since the INSM-specific siRNA inhibited the growth of HSV-1, we conclude that INSM1 induction plays a positive role in viral replication. It is well known that ICP0 is sufficient to trigger HSV reactivation in latently infected trigeminal ganglion neurons. INSM1 may be involved as a host factor in the reactivation of HSV latency. We are now investigating the roles of INSM1 in the HSV life cycle in a mouse model system.

## Methods

### Cells and viruses

Vero cells derived from a stable African monkey kidney were propagated in Eagle's minimal essential medium (EMEM) containing 10% calf serum. HEp-2 cells derived from a human laryngeal carcinoma were propagated in Dulbecco's modified Eagle medium (DMEM) containing 10% fetal calf serum (FCS). HaCaT, a human keratinocyte cell line, was kindly provided by Dr. Norbert E Fusenig (German Cancer Resarch Center, Heidelberg, Germany) [40]. HaCaT cells were propagated in DMEM containing 5% FCS. SBC-3 cells derived from a human small-cell lung carcinoma were propagated in EMEM containing 10% FCS (provided by Health Sciences Research Resources Bank, Japan). A primary culture of NHEK of neonatal foreskin (NHEK(F)) was obtained from Kurabo (Osaka, Japan) and subcultured in Humedia-KB2 medium (Kurabo) supplemented with bovine pituitary extract (0.4% v/v), human epidermal growth factor (0.1 ng/ml), insulin (10 μg/ml), hydrocortisone (0.5 μg/ml), gentamycin (50 μg/ml), and amphotericin B (50 ng/ml) (Complete Humedia-KG2). All viruses were propagated and titered on Vero cells. The WT HSV-1 strain F and its ΔUS3 mutant R7041 were kindly provided by B. Roizman, University of Chicago. The properties of the WT HSV-2 strain 186 and its ΔUS3 mutant L1BR1 have been described [41]. Viruses were propagated in Vero cells by infection at a low MOI (0.01 PFU/cell), and infected cells and growth medium were harvested together when almost all cells showed cytopathic effects. Virus stocks were prepared by one cycle of freezing and thawing followed by centrifugation at 3,000 rpm for 5 min at 4°C to remove cell debris, and then stored at -80°C. Virus stock titers were determined on Vero cells by plaque assay. Subconfluent NHEK cells plated in 10-cm dishes were mock-infected or infected with HSV-1 (F, R7041) or HSV-2 (186, L1BR1) (3 PFU/cell) for 60 min at 37°C, followed by the addition of DMEM containing 1% FCS.

### RNA isolation

At the indicated times after infection, the medium was removed and cells were washed with PBS and then lysed with ISOGEN reagent (Nippon Gene Co., Toyama, Japan). Lysates were stored at -80°C. RNA for microarray analysis was isolated with Isogen and purified using the RNeasy MinElute kit (Qiagen, Valencia, CA, USA) according to the manufacturer's instructions. RNA quality was assessed using an Agilent 2100 Bioanalyzer (Agilent Technologies, Palo Alto, CA). Double-stranded cDNA was synthesized using a T7-oligo (dT) primer and the One-cycle cDNA synthesis kit (Affymetrix Inc., Santa Clara, CA) and subsequently purified using a Sample Cleanup Module (Affymetrix).

### Microarray data analysis

Hybridization samples were prepared and processed according to the GeneChip Expression Analysis Technical Manual, 701021 Rev. 5. The Human Genome U133 plus 2.0 chips comprise 54,765 probe sets. Data were analyzed using the GeneChip Operating Software version 1.4 (Affymetrix 690036) according to the GeneChip Expression Analysis Data Analysis Fundamentals. Using DNA MicroArray Viewer (Kurabo, Osaka, Japan), fold changes in expression between each of the infected samples compared with mock-infected controls of the same cell type were calculated (log_2 _transformed) and further classified as unchanged, increased (signal log ratio change *P *value of < 0.005), decreased (signal log ratio change *P *value of > 0.995), or marginally increased or decreased. Sequences that showed differential expression in infected cells were grouped according to the GeneOntology terms for biological processes, available on the National Center for Biotechnology Information website (March 2006, NCBI Build 36.1). Sequences not yet annotated by GeneOntology were not analyzed further. Genes that could be placed into more than 1 group according to these annotations were arbitrarily assigned to a single group.

### RT-PCR analysis

INSM1 mRNA levels were determined by RT-PCR. To ensure the absence of genomic DNA contamination, the samples were tested by PCR without RT. First-strand cDNA was prepared from 1 μg total RNA using the Promega reverse transcription system (Promega, Madison, Wisconsin, USA, A3500), and cDNA was used as a PCR template for TaKaRa Ex Taq (TAKARA BIO INC, Shiga, Japan, RR001A). The PCR conditions were as follows: 98°C for 10 s, 58°C for 30 s, and 72°C for 30 s. The primers used were as follows: human INSM1, (forward) 5′-AACTGTGCCTTCGCTTGGA-3′ and (reverse) 5′-ACGAGACAAACGCGTACAGCT-3′ (269-bp product); human GAPDH, (forward) 5′-CGGAGTCAACGGATTTGGTCGTAT-3′ and (reverse) 5′-AGCCTTCTCCATGGTGGTGAAGAC-3′ (307-bp product).

### Plasmids and constructs

Human INSM1 cDNA was amplified from SBC3 cells by PCR using specific primers (forward, 5′- GCCGGATCCAACATGCCCCGCGGCTTCCTGGTGAA -3′; reverse, 5′- CGGAATTCCAGCAGGCCGGGCGCACGGG-3′) and KOD FX (TOYOBO, Osaka, Japan, KFX-101). The PCR conditions were as follows: denaturation at 94°C for 2 min, 35 cycles of denaturation at 98°C for 10 s, and extension at 68°C for 1 min 30 s. The PCR products were electrophoresed on a 1.5% agarose gel, and fragments were extracted using a QIAquick Gel Extraction kit (Qiagen). These extracted fragments were amplified by PCR under the abovementioned conditions using specific primers containing *BamHI *and *EcoRI *sites. The PCR products were digested with *BamHI *and *EcoRI *and cloned into the respective cognate sites of pEF-mycHisB (Invitrogen, Carlsbad, CA, USA). cDNA fragments of INSM1 mutants were amplified from full-length. pEF-INSM1-C was constructed using PCR on this template with primers (forward, 5′-CGGGATCCAACATGGAGGGCCCGGTGGAG-3′ and reverse, 5′- CGGAATTCCAGCAGGCCGGGCGCACGGG-3′) followed by substitution of a *BamHI*- and *EcoRI*-restricted amplified fragment. pEF-INSM1-N was constructed using PCR with a reverse primer 5′-CGGAATTCACCGGGCCCTCCTTGAT-3′ to delete INSM1-C. The VP16 expression vector pCDNA-HSV-1 UL48 has been described [42]. The complete coding sequences of all constructs were verified by sequencing.

### Transfection and infection

Cells were plated on 35-mm dishes and incubated for 24 h before transfection or infection. Cells were transfected with 1 μg of each plasmid DNA using FugeneHD (Roche Applied Science, Indianapolis, IN, USA, 04709705001) according to the manufacturer's recommendations. In some experiments, transfected cells were also infected with HSV-1(F) 20-24 h after transfection. Infections were performed by exposing cells to a minimal volume of virus diluted at MOI of 3 PFU/cell in EMEM without FCS. After 1 h of adsorption, the virus inoculum was replaced with DMEM containing 1% FCS and cells were incubated for the indicated time periods.

### Immunofluorescence confocal microscopy

Indirect immunofluorescence confocal microscopy was performed as previously described with modifications [42]. In brief, cells grown on cover slips were fixed in 4% paraformaldehyde in PBS for 20 min and permeabilized with 0.5% Triton X-100 for 5 min at room temperature. Coverslips were incubated for 1 h at 37°C sequentially with 20% normal goat serum (DAKO, Glostrup, Denmark) and primary and secondary antibodies (30 min for each). The following primary antibodies were used: polyclonal anti-Myc (1:100 dilution; Santa Cruz Biotechnology, Inc., sc-40) and anti-INSM1 (1:100 dilution; Abcam, ab30940) antibodies; monoclonal anti-ICP0 (1:100 dilution; Virusys, H1A027-100) and anti-ICP8 (1:50 dilution; Abcam, ab20194) antibodies. FITC- or TRITC-conjugated goat anti-rabbit and anti-mouse antibodies were used as secondary antibodies. In some experiments, coverslips were additionally incubated with DRAQ5™ (1:4,000, Biostatus, Leicestershire, UK) for 30 min at room temperature to stain nuclear DNA together with secondary antibody staining. For double staining with two different mouse monoclonal antibodies, mouse anti-Myc and anti-ICP0 or anti-ICP8 antibodies were directly conjugated with AlexaFluor 488 and 555, respectively, using the Zenon Mouse IgG1 labeling kit (Molecular Probes, Eugene, OR, Z25002 and Z25005) according to the manufacturer's protocol, and cells were incubated sequentially with each antibody. Confocal images were captured using the Zeiss LSM510 system (Carl Zeiss, Jena, Germany). Images were acquired and processed using Adobe Photoshop Elements 7.

### Luciferase Reporter Assay

To generate luciferase reporter plasmids of the human INSM1 promoter, PCR fragments (-441 to +26 bp) from the HEp-2 genome were inserted into the *KpnI *and *XhoI *sites of the pGL3 basic luciferase expression vector (Promega, #E1751). To generate luciferase reporter plasmids of the HSV-1 ICP0 promoter, PCR fragments (-834 to +145 bp) from the HSV-1(F)-infected HEp-2 genome were inserted into the *KpnI *and *XhoI *sites of the pGL3 basic luciferase expression vector. Using FugeneHD, HEp-2 or HaCaT cells were transfected with luciferase reporter plasmids and INSM1 expression plasmids according to the manufacturer's instructions. The cells were plated in 48-well plates and transfected with expression vectors and a reporter gene. HEp-2 or HaCaT cells in 48-well plates were transfected into cells at 30-40% confluence. Triplicate wells received 100 ng of reporter plasmid and 5-50 ng of expression vector DNAs, and cells were harvested for luciferase assays after 24 h. HEp-2 or HaCaT cells were cultured in 48-well microplates and transfected with 100 ng of hINSM1-Luc or ICP0-Luc reporter plasmids and the desired expression plasmids (5-50 ng of each expression plasmid). The total amount of plasmid DNA was kept constant by balancing with the empty vector. Transfection mixtures were added dropwise into cell culture medium and incubated at 37°C for 48 h. Transfected cells were harvested in PBS and lysed with 50-70 μl of 1 × passive lysis buffer (Promega, E1941). Cell lysates were clarified by centrifugation and assayed for luciferase activity using the luciferase assay system (Promega, E1483). Luciferase activity was normalized by protein concentration of each sample, which was determined by the Bio-Rad Protein assay kit (BIO-RAD, #500-0006).

### ChIP assay

Forty-eight hours after transfection, transfectants were infected with HSV-1(F) at MOI of 3 PFU/cell 7 h post-infection. The cells were fixed by adding formaldehyde to the medium (1% final concentration) for 15 min at room temperature. The cross-linking reaction was terminated by adding glycine to a final concentration of 0.125 M. The fixed cells were washed twice in ice-cold PBS and solubilized in SDS lysis buffer (1% SDS, 10 mM EDTA, 50 mM Tris-HCl, pH 8.0) containing protease inhibitor cocktail (Sigma-Aldrich), and 10 μM PMSF for 20 min on ice. The chromatin complexes were sheared by sonication to an average fragment size of 200-500 bp. Sonication was performed with Bioruptor (Cosmo bio co., Ltd., Tokyo, Japan), using 100 μl in 1.5 ml tube. One time of sonication was done with 30 s on and 30 s off with high power. Ten times of sonication were performed with 10 min. ChIP assays were performed using the OneDay ChIP assay kit (Diagenode, Nippon Gene 313-86401) according to the manufacturer's protocol. Cells were lysed in SDS lysis buffer (1% SDS, 10 mM EDTA, 50 mM Tris-HCl, pH 8.0) containing protease inhibitor cocktail (Sigma-Aldrich) and 10 μM PMSF and then sonicated. Chromatin solution was subjected to immunoprecipitation using a OneDay ChIP kit (Diagenode, Sparta, NJ) according to the manufacturer's instructions. The following antibodies were used: anti-Myc (Santa Cruz Biotechnology, Inc., sc-789) and negative control rabbit IgG (contained in the OneDay ChIP assay kit). The HSV-1 ICP0 promoter was amplified by PCR using the following primers: (forward) 5′-AAGCCCCTGTTGCGTCGGCG-3′ and (reverse) 5′-TTATCCCCACGCCTTTCCC-3′ (234 bp). As a negative control, the HSV-1 ICP27 promoter was amplified using the following primers: 5′-GGGGTACCCCCAACGACCCCGCCCATGGG-3′ and 5′-GGCTCGAGGGGGTGTCGGATATCGCCTCT-3′ (396 bp).

### Gene Silencing

To knock down INSM1 expression, HEp-2 cells were transfected with an INSM1-specific siRNA (5′-UCCGCAAGCUGCACUUCGATT-3′) or a scrambled (5′-GCAUUCCAUCGCGCGGUCACATT-3′) (20 μM; Nippon EGT Co., Ltd.) ribooligonucleotide using X-tremeGENE siRNA transfection reagent (Roche, #04476093001). At 24 h after siRNA transfection, HEp-2 cells were infected with HSV-1(F) at MOI of 1, and total RNA was extracted 6 h after infection and analyzed by RT-PCR. At 24 h posttransfection, HEp2 cells were infected with HSV-1 at MOI of 0.01 PFU/cell at 24 h. Total protein lysate or culture medium were collected and analyzed by western blotting or virus titration, respectively.

### Statistics

Data were analyzed by Student's *t *test. *P *< 0.05 was considered significant.

## Competing interests

The authors declare that they have no competing interests.

## Authors' contributions

MK and YN designed the research, MK, FG, and CL performed the experimental work, MK conducted data analysis and drafted the manuscript, and FG, HK, and YN participated in data analysis and review of the manuscript. All authors read and approved the final manuscript.
